# Supplementary material for: Severe COVID-19 in pregnancy has a distinct serum profile, including greater complement activation and dysregulation of serum lipids
Source: PLoS One. 2022 Nov 16;17(11):e0276766. doi: 10.1371/journal.pone.0276766 (PMC9668183; doi:10.1371/journal.pone.0276766)
Supplement: S1 Fig — (PDF) [file pone.0276766.s003.pdf]

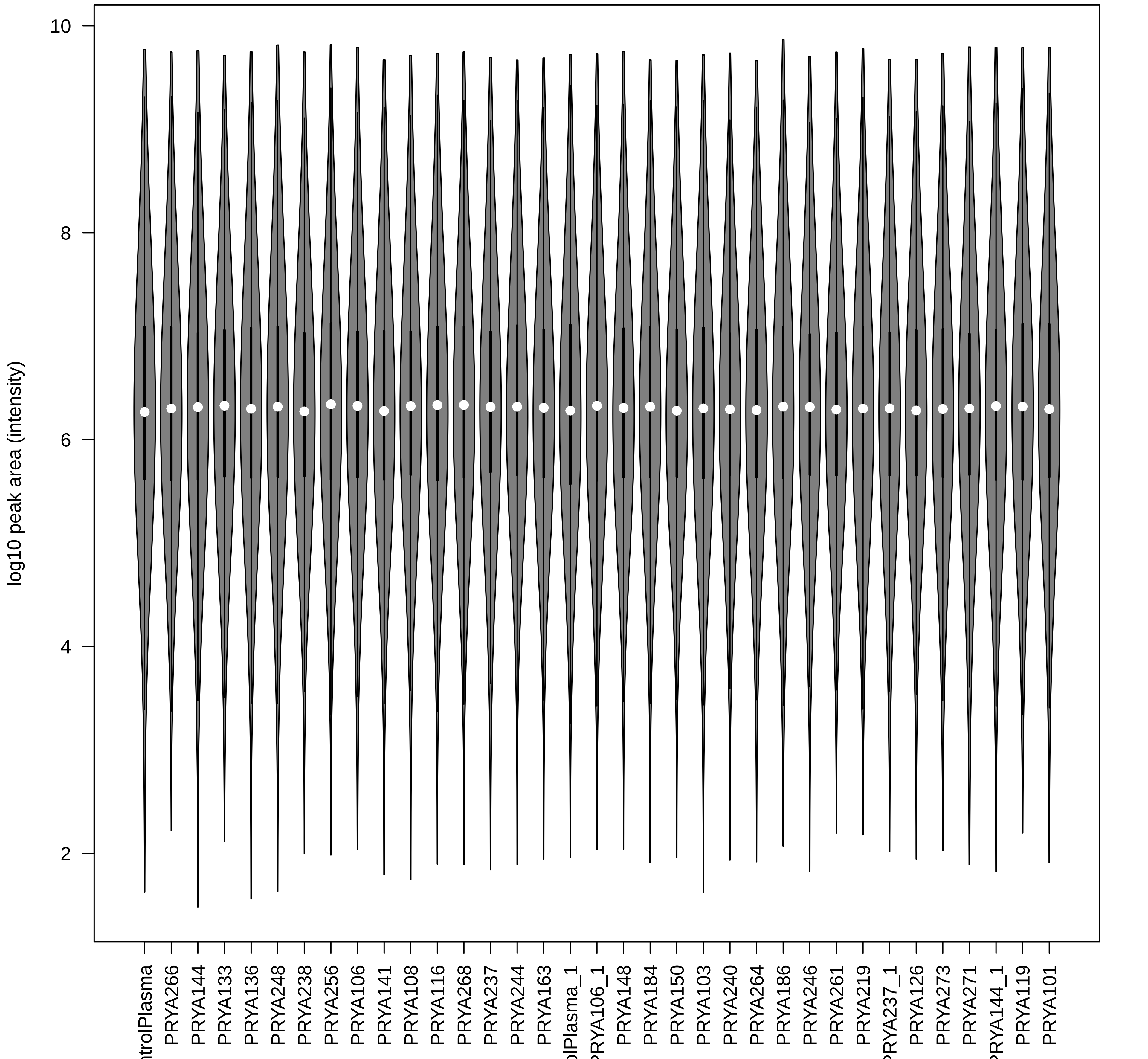

log10 peak area (intensity)
10
8
6
4
2
ntrolPlasma
PRYA266
PRYA144
PRYA133
PRYA136
PRYA248
PRYA238
PRYA256
PRYA106
PRYA141
PRYA108
PRYA116
PRYA268
PRYA237
PRYA244
PRYA163
olPlasma_1
PRYA106_1
PRYA148
PRYA184
PRYA150
PRYA103
PRYA240
PRYA264
PRYA186
PRYA246
PRYA261
PRYA219
PRYA237_1
PRYA126
PRYA273
PRYA271
PRYA144_1
PRYA119
PRYA101

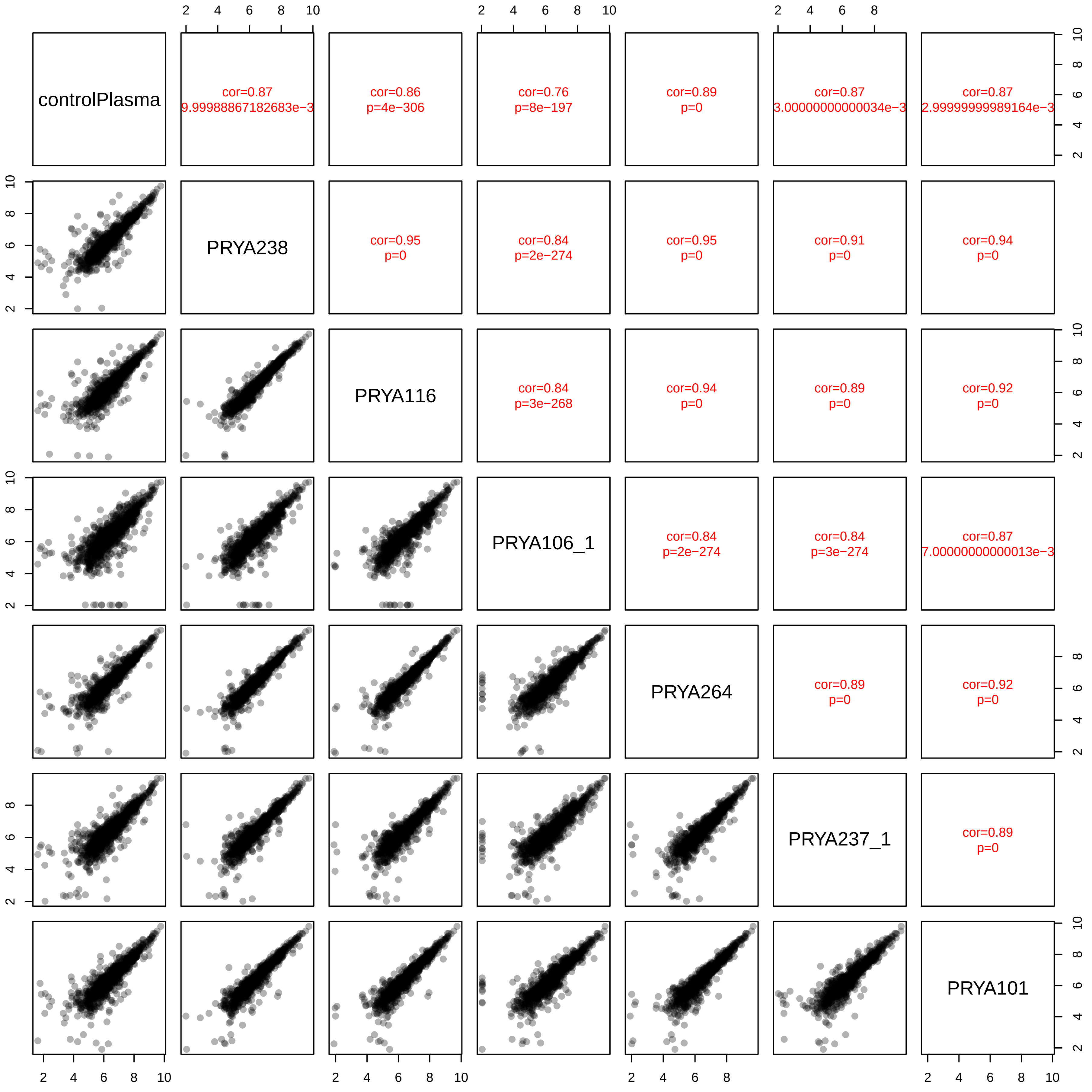
controlPlasma
PRYA238
PRYA116
PRYA106_1
PRYA264
PRYA237_1
PRYA101
cor=0.87
9.99988867182683e−3
cor=0.86
p=4e−306
cor=0.76
p=8e−197
cor=0.89
p=0
cor=0.87
3.00000000000034e−3
cor=0.87
2.99999999989164e−3
cor=0.95
p=0
cor=0.84
p=2e−274
cor=0.95
p=0
cor=0.91
p=0
cor=0.94
p=0
cor=0.84
p=3e−268
cor=0.94
p=0
cor=0.89
p=0
cor=0.92
p=0
cor=0.84
p=2e−274
cor=0.84
p=3e−274
cor=0.87
7.00000000000013e−3
cor=0.89
p=0
cor=0.92
p=0
cor=0.89
p=0

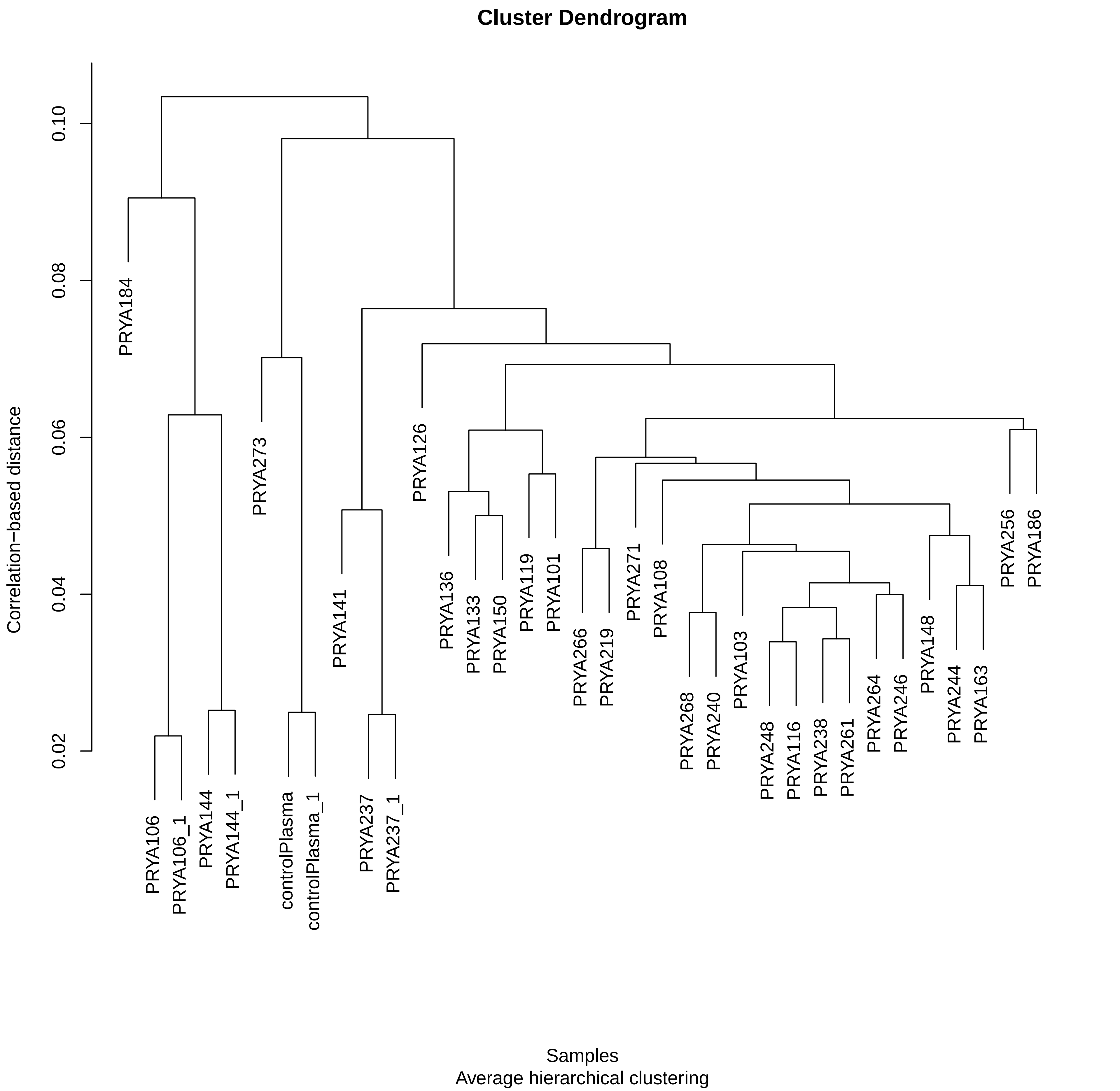
Cluster Dendrogram
Correlation−based distance
0.02
0.04
0.06
0.08
0.10
PRYA184
PRYA106
PRYA106_1
PRYA144
PRYA144_1
PRYA273
controlPlasma
controlPlasma_1
PRYA141
PRYA237
PRYA237_1
PRYA126
PRYA136
PRYA133
PRYA150
PRYA119
PRYA101
PRYA266
PRYA219
PRYA271
PRYA108
PRYA268
PRYA240
PRYA103
PRYA248
PRYA116
PRYA238
PRYA261
PRYA264
PRYA246
PRYA148
PRYA244
PRYA163
PRYA256
PRYA186
Samples
Average hierarchical clustering

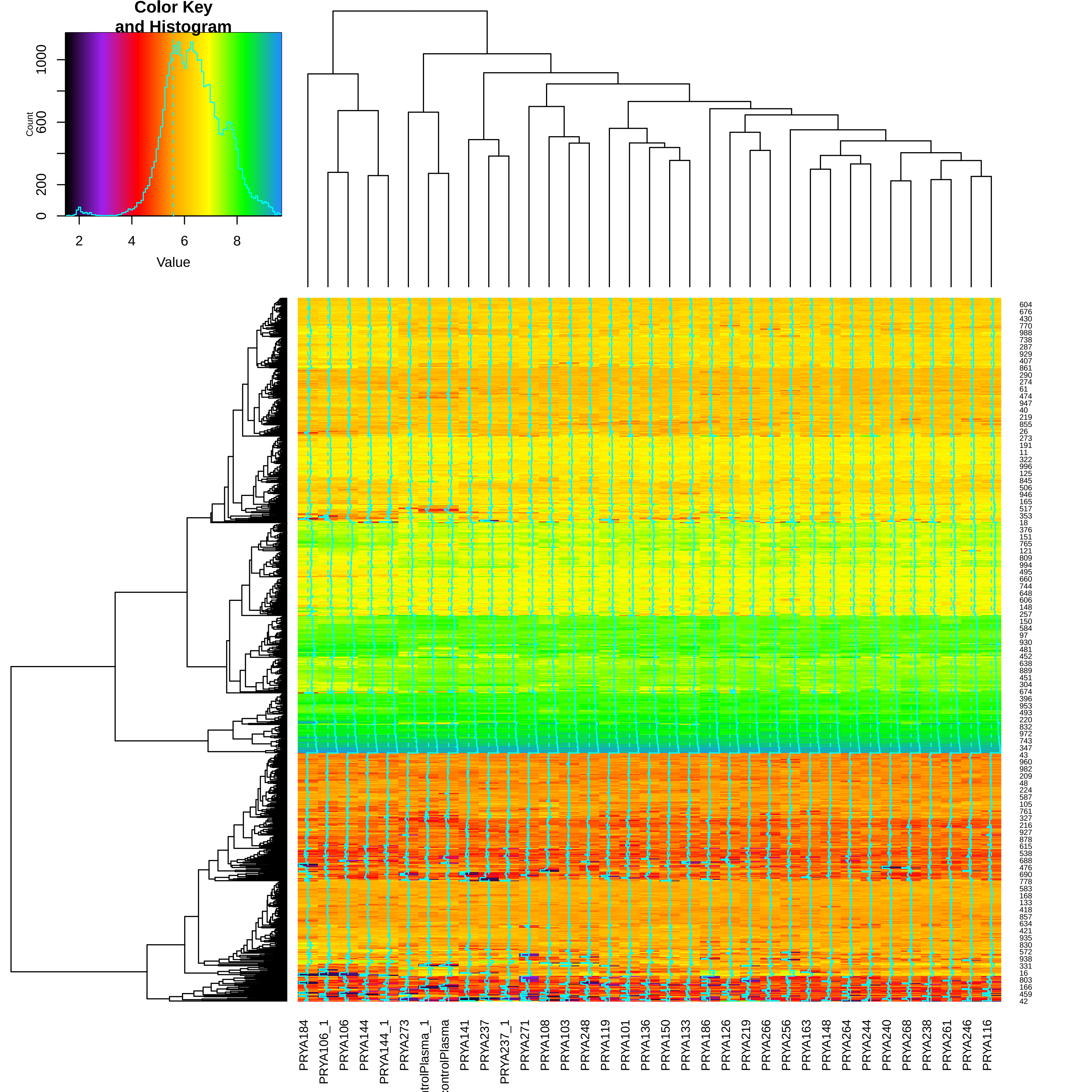

Color Key
and Histogram
Count
0
200
600
1000
2
4
6
8
Value
604
676
430
770
988
738
287
929
407
861
290
274
61
474
947
40
219
855
26
273
191
11
322
996
125
845
506
946
165
517
353
18
376
151
765
121
809
994
495
660
744
648
606
148
257
150
584
97
930
481
452
638
889
451
304
674
396
953
493
220
832
972
743
347
43
960
982
209
48
224
587
105
761
327
216
927
878
615
538
688
476
690
778
583
168
133
418
857
634
421
935
830
572
938
331
16
803
166
459
42
PRYA184
PRYA106_1
PRYA106
PRYA144
PRYA144_1
PRYA273
trolPlasma_1
ontrolPlasma
PRYA141
PRYA237
PRYA237_1
PRYA271
PRYA108
PRYA103
PRYA248
PRYA119
PRYA101
PRYA136
PRYA150
PRYA133
PRYA186
PRYA126
PRYA219
PRYA266
PRYA256
PRYA163
PRYA148
PRYA264
PRYA244
PRYA240
PRYA268
PRYA238
PRYA261
PRYA246
PRYA116

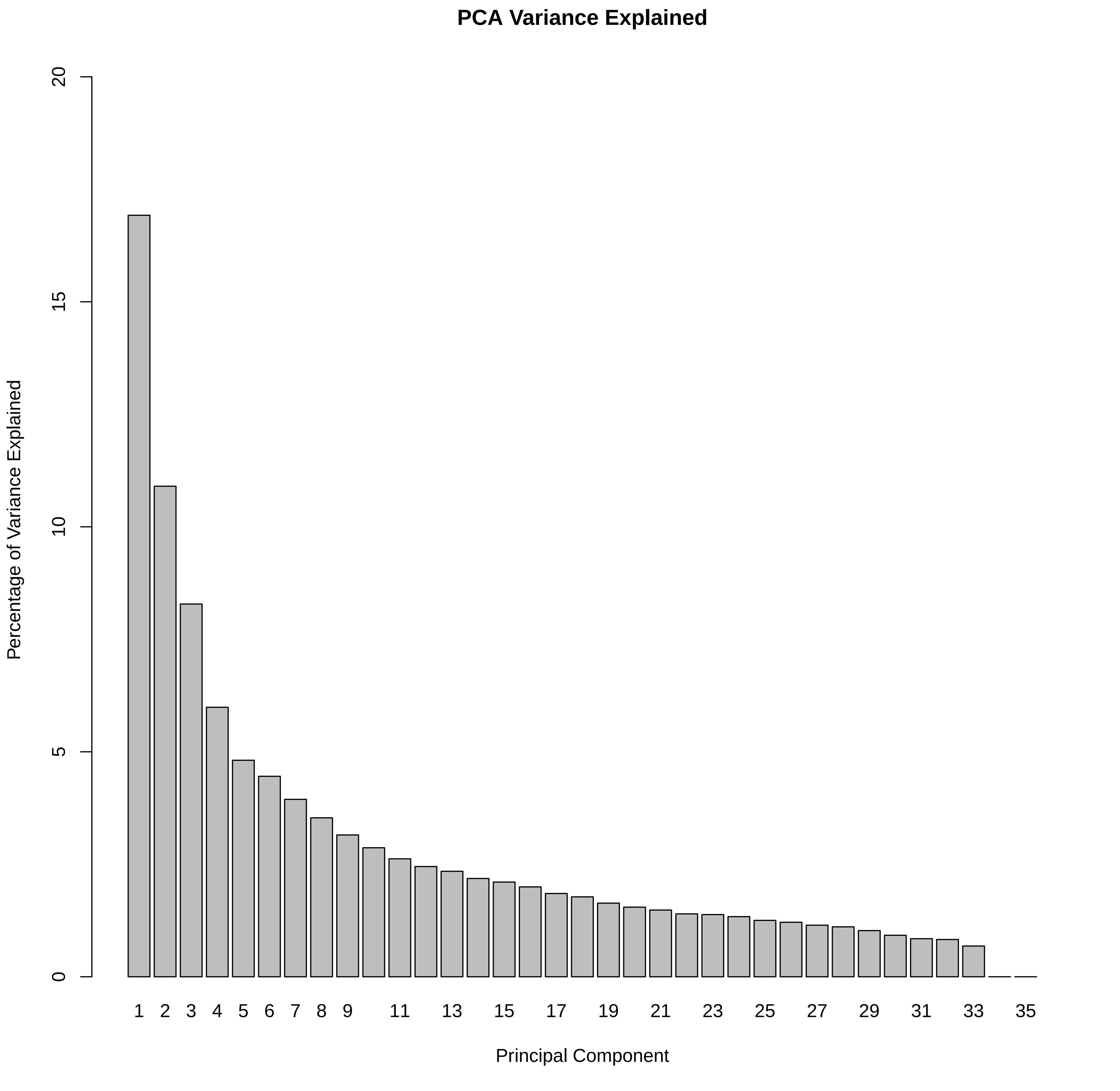

PCA Variance Explained
Percentage of Variance Explained
0
5
10
15
20
1
2
3
4
5
6
7
8
9
11
13
15
17
19
21
23
25
27
29
31
33
35
Principal Component

PCA plot

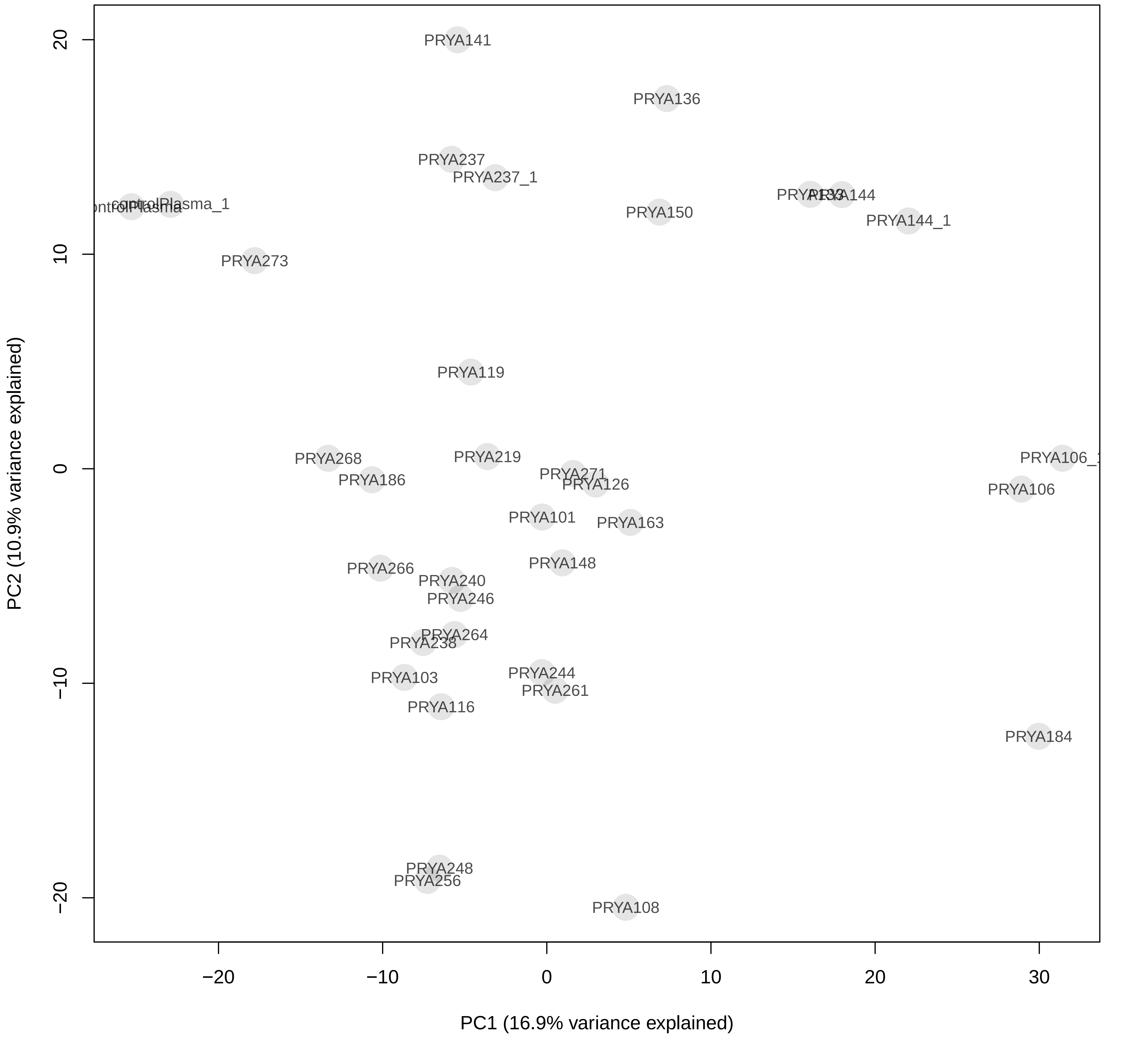

PRYA141
PRYA136
PRYA237
PRYA237_1
controlPlasma_1
PRYA150
PRYA144
PRYA144_1
PRYA273
PRYA119
PRYA268
PRYA186
PRYA219
PRYA271
PRYA126
PRYA106
PRYA101
PRYA163
PRYA148
PRYA266
PRYA240
PRYA246
PRYA264
PRYA238
PRYA244
PRYA261
PRYA103
PRYA116
PRYA184
PRYA248
PRYA256
PRYA108
PC1 (16.9% variance explained)
PC2 (10.9% variance explained)

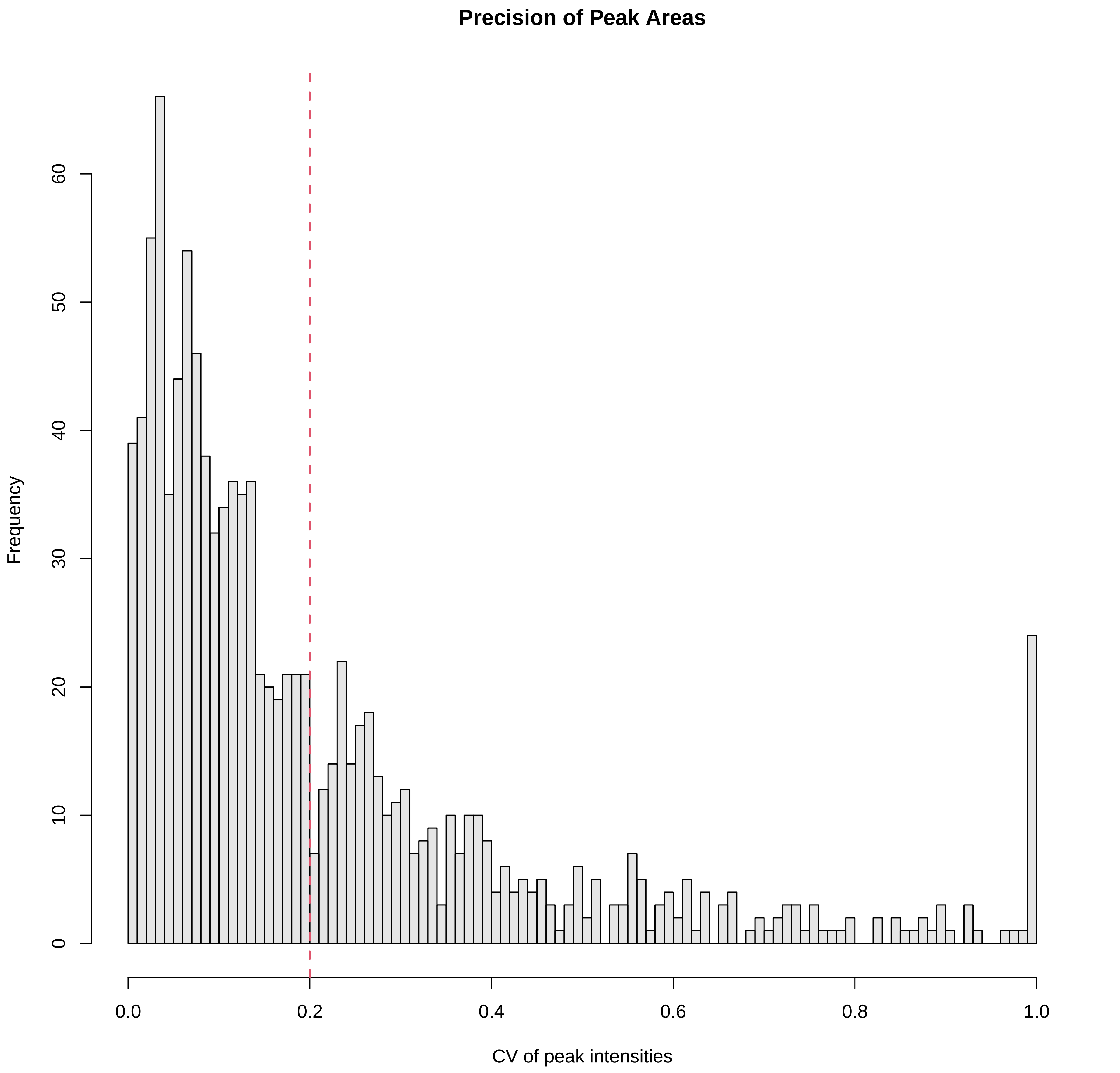
Precision of Peak Areas
Frequency
0
10
20
30
40
50
60
0.0
0.2
0.4
0.6
0.8
1.0
CV of peak intensities

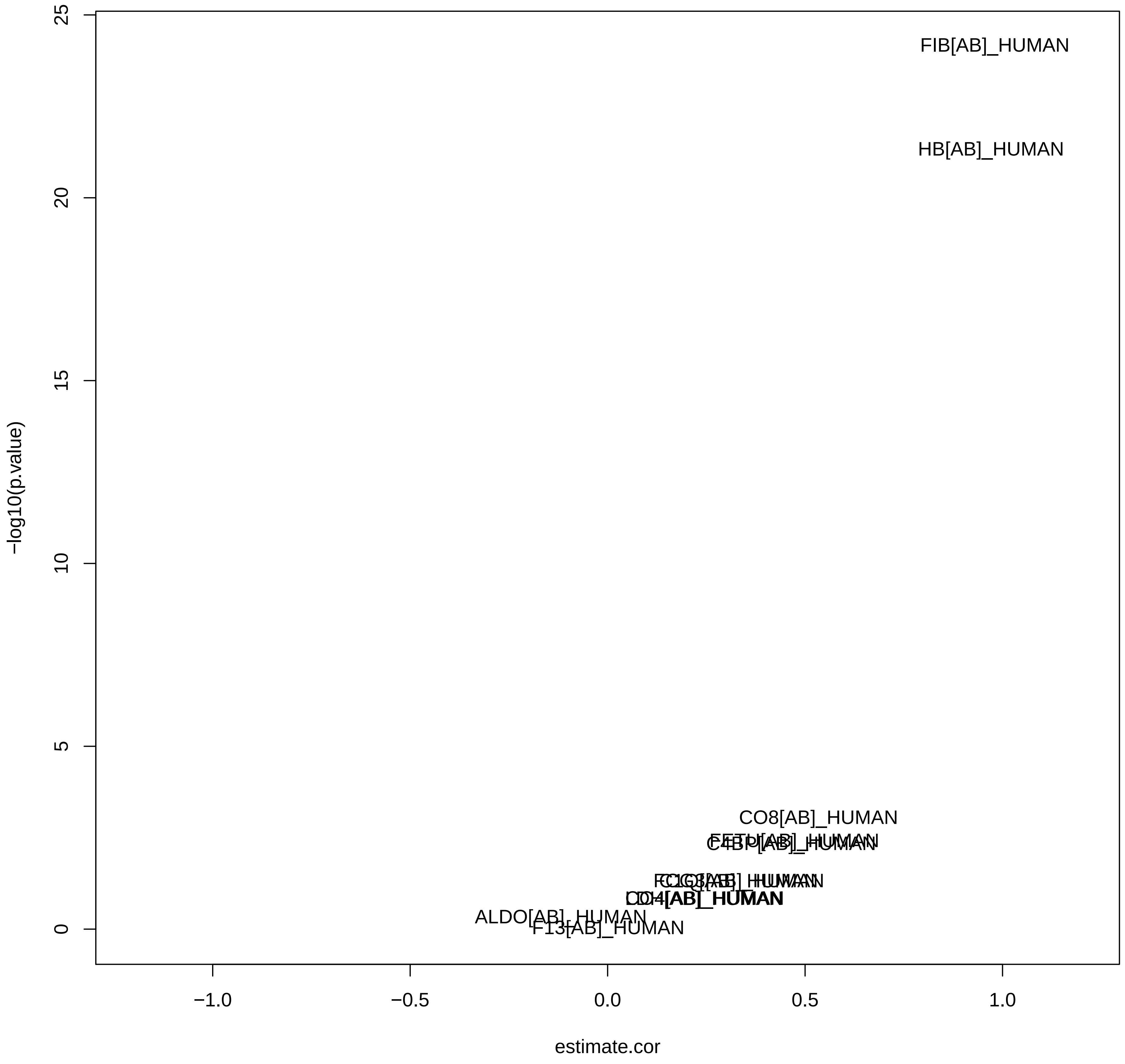

FIB[AB]_HUMAN
HB[AB]_HUMAN
CO8[AB]_HUMAN
C4BP[AB]_HUMAN
ALDO[AB]_HUMAN
F13[AB]_HUMAN
25
20
15
10
5
0
−log10(p.value)
−1.0
−0.5
0.0
0.5
1.0
estimate.cor

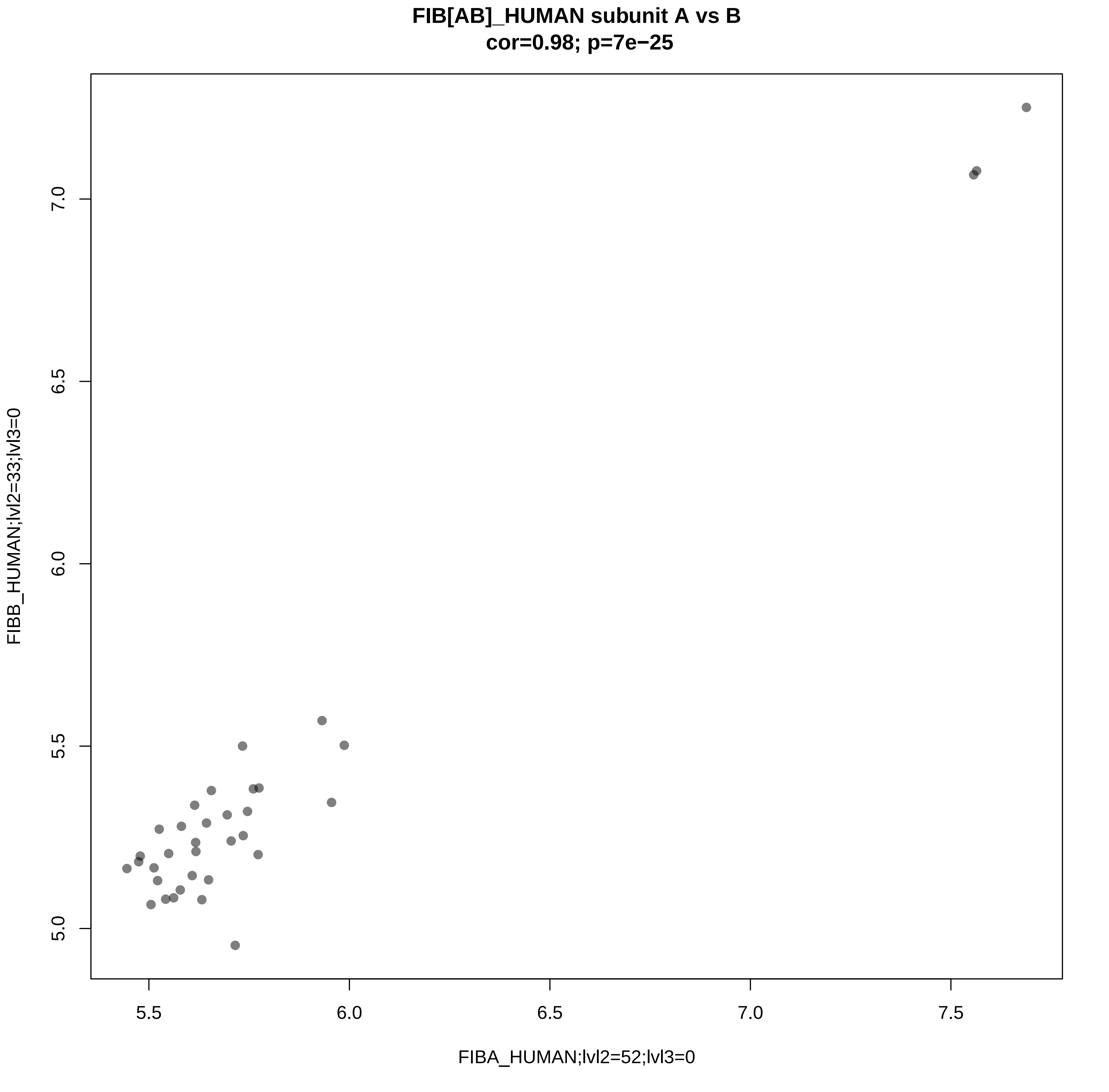
FIB[AB]_HUMAN subunit A vs B
cor=0.98; p=7e−25
FIBB_HUMAN;lvl2=33;lvl3=0
FIBA_HUMAN;lvl2=52;lvl3=0
5.0
5.5
6.0
6.5
7.0
5.5
6.0
6.5
7.0
7.5

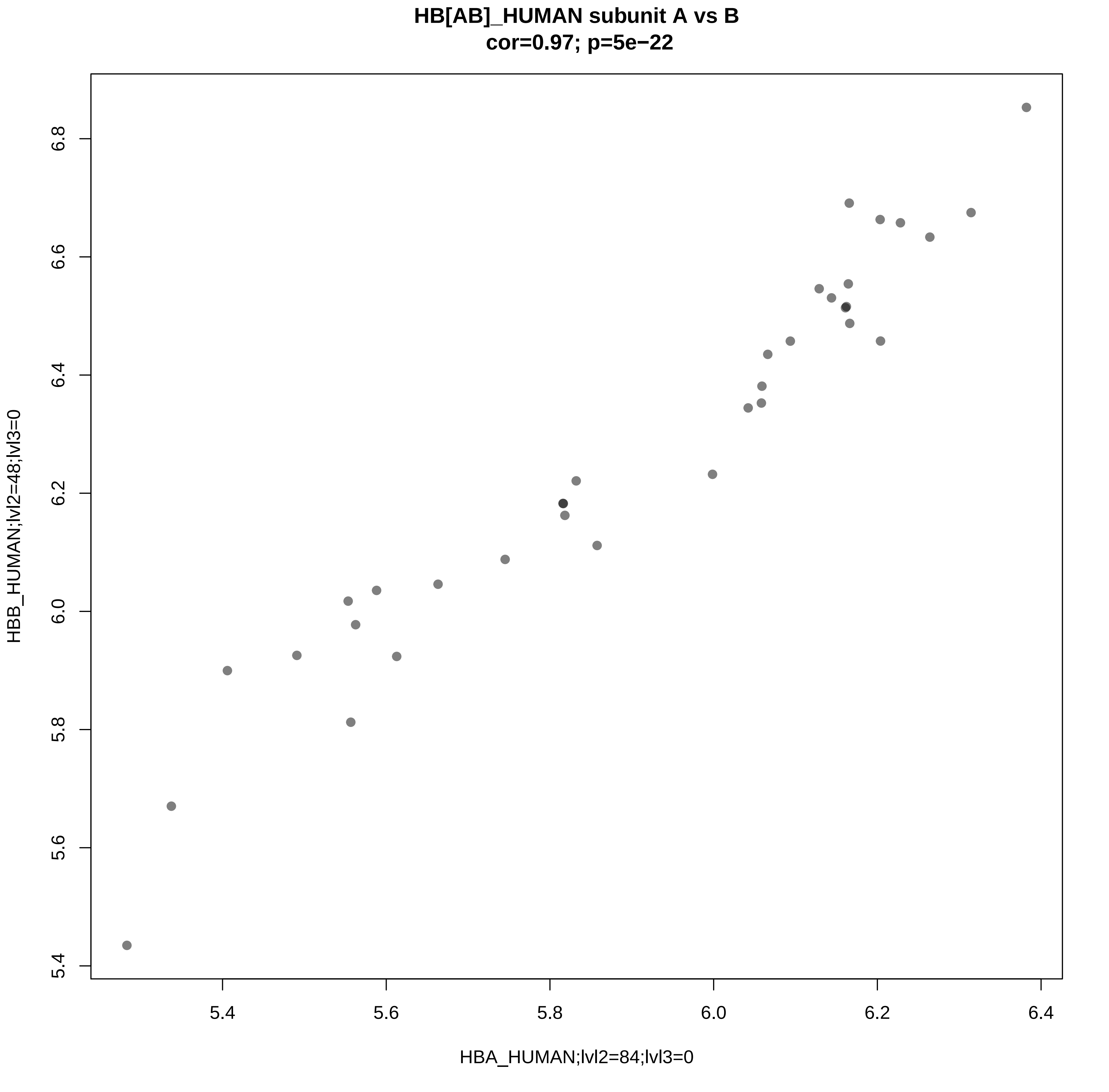
HB[AB]_HUMAN subunit A vs B
cor=0.97; p=5e−22
HBB_HUMAN;lvl2=48;lvl3=0
HBA_HUMAN;lvl2=84;lvl3=0
5.4
5.6
5.8
6.0
6.2
6.4
6.6
6.8

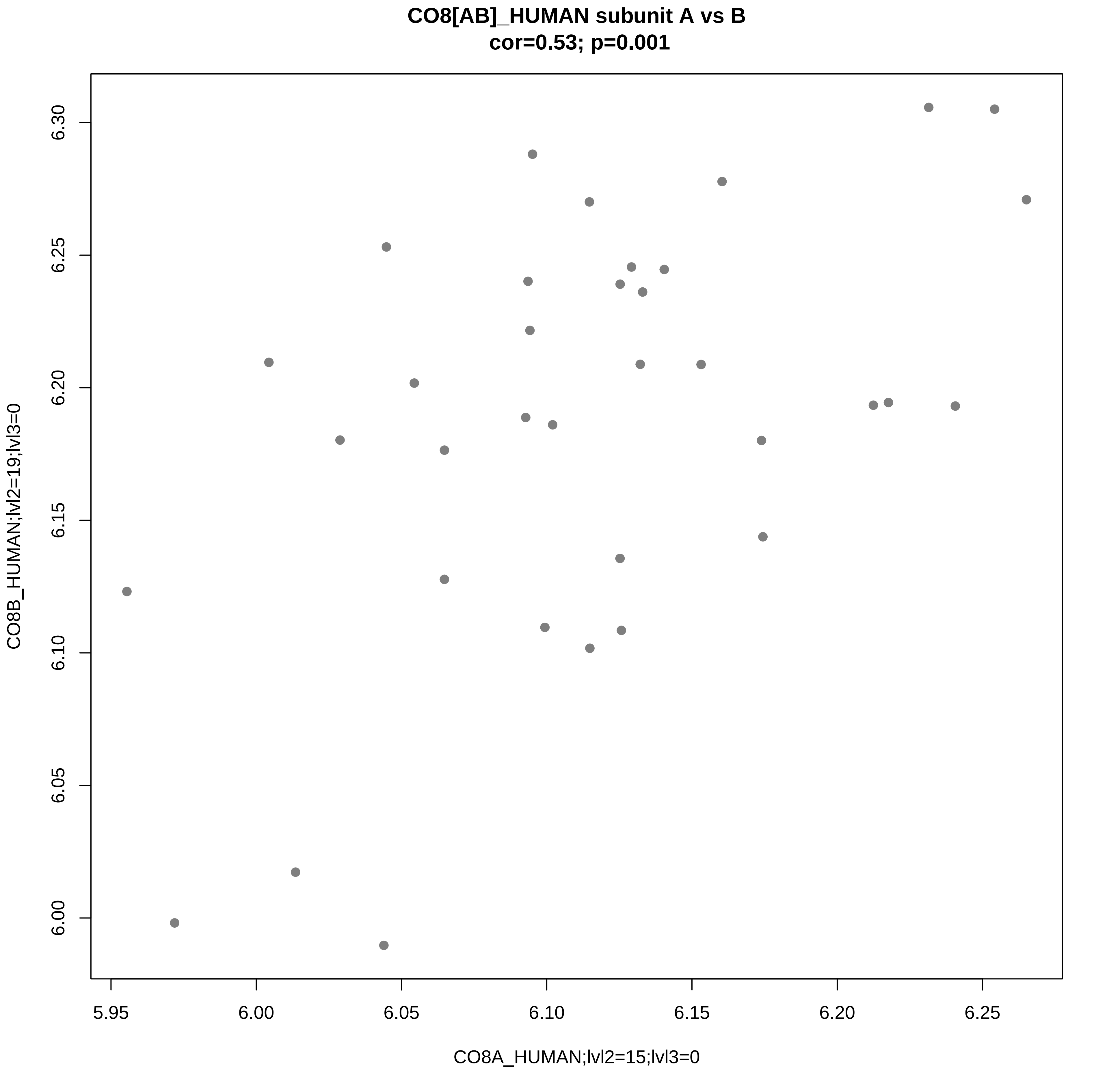
CO8[AB]_HUMAN subunit A vs B
cor=0.53; p=0.001
CO8B_HUMAN;lvl2=19;lvl3=0
CO8A_HUMAN;lvl2=15;lvl3=0
6.00
6.05
6.10
6.15
6.20
6.25
6.30
5.95
6.00
6.05
6.10
6.15
6.20
6.25

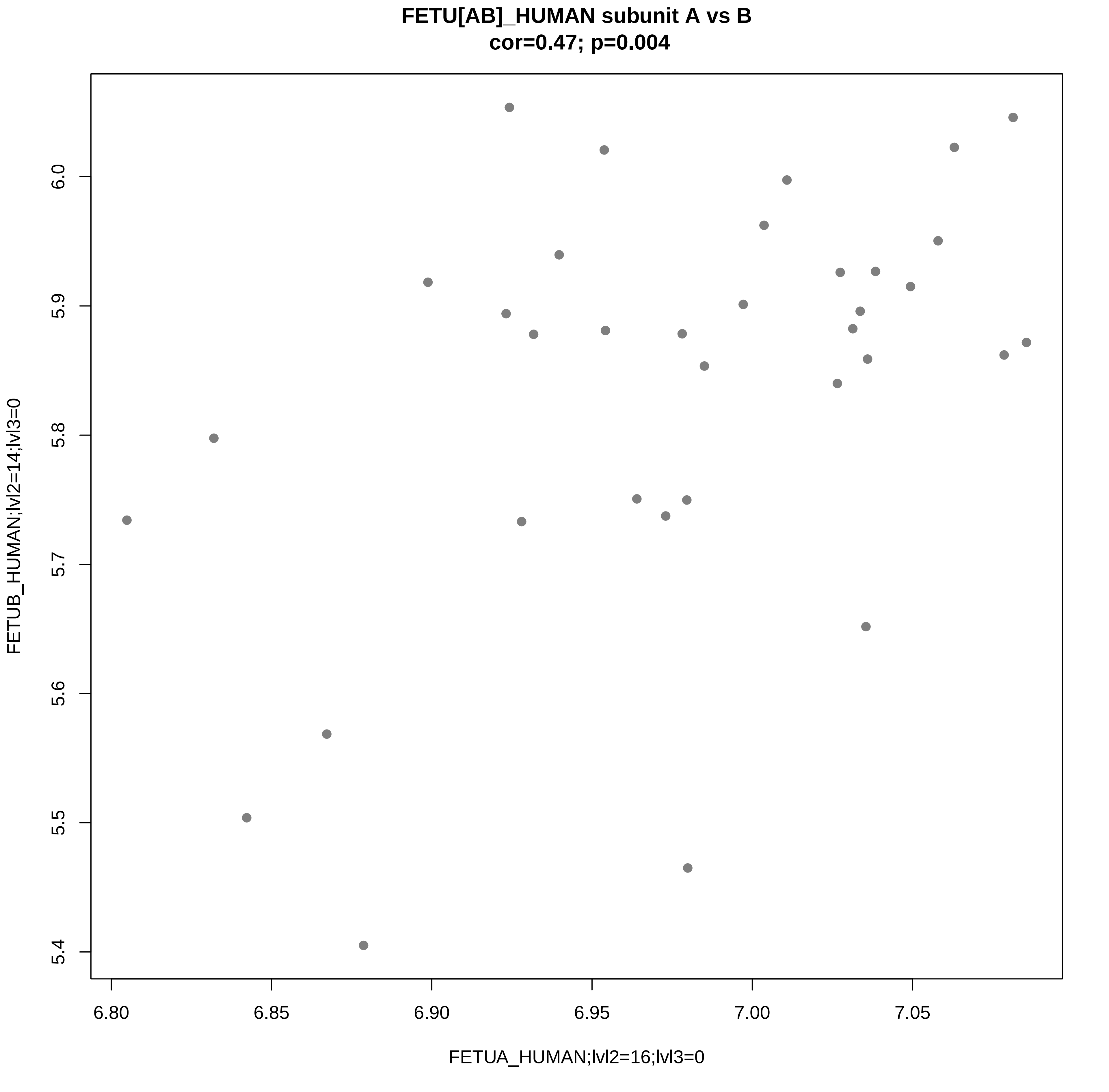
FETU[AB]_HUMAN subunit A vs B
cor=0.47; p=0.004
FETUB_HUMAN;lvl2=14;lvl3=0
6.0
5.9
5.8
5.7
5.6
5.5
5.4
6.80
6.85
6.90
6.95
7.00
7.05
FETUA_HUMAN;lvl2=16;lvl3=0

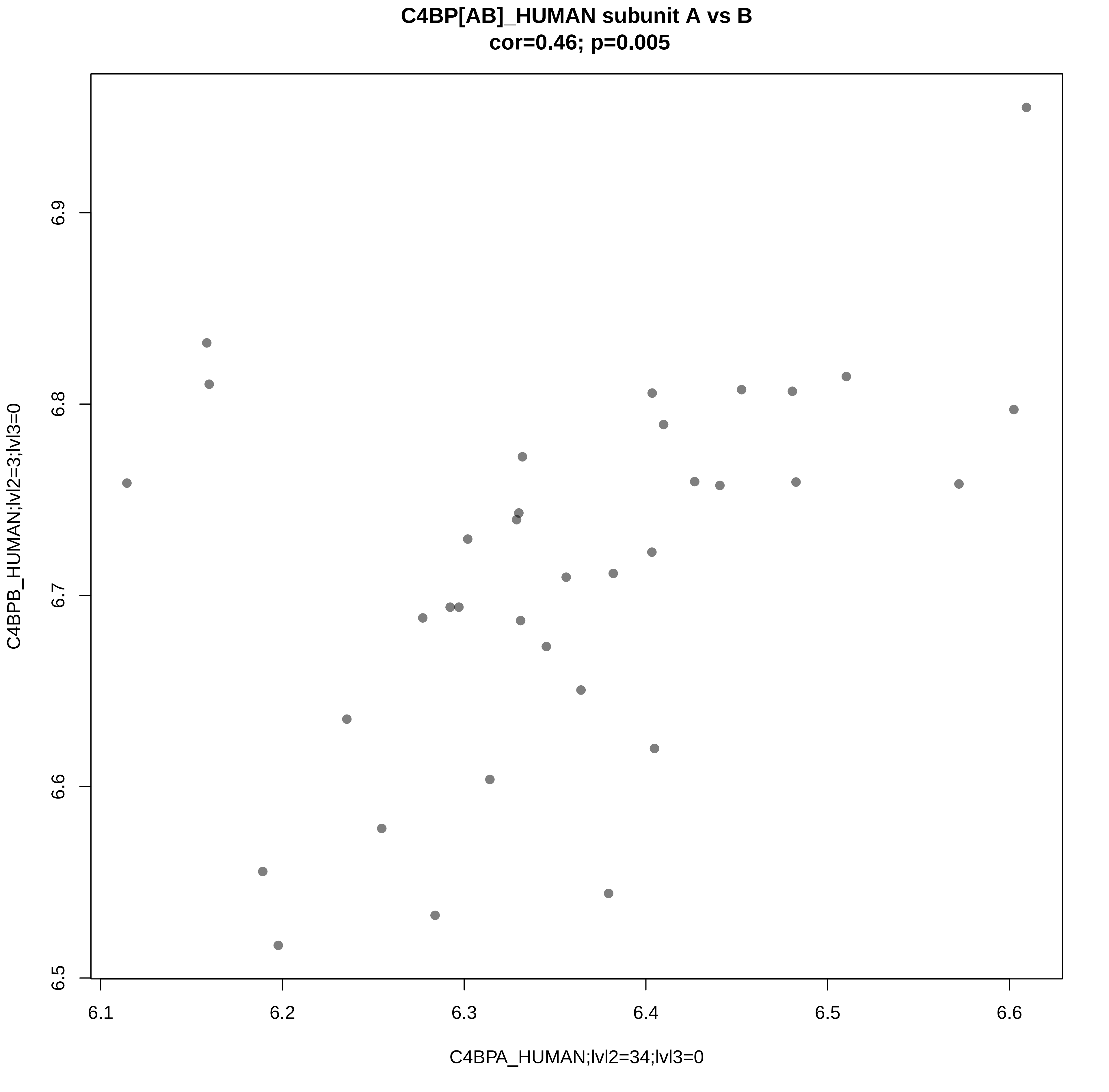
C4BP[AB]_HUMAN subunit A vs B
cor=0.46; p=0.005
C4BPB_HUMAN;lvl2=3;lvl3=0
6.5
6.6
6.7
6.8
6.9
6.1
6.2
6.3
6.4
6.5
6.6
C4BPA_HUMAN;lvl2=34;lvl3=0

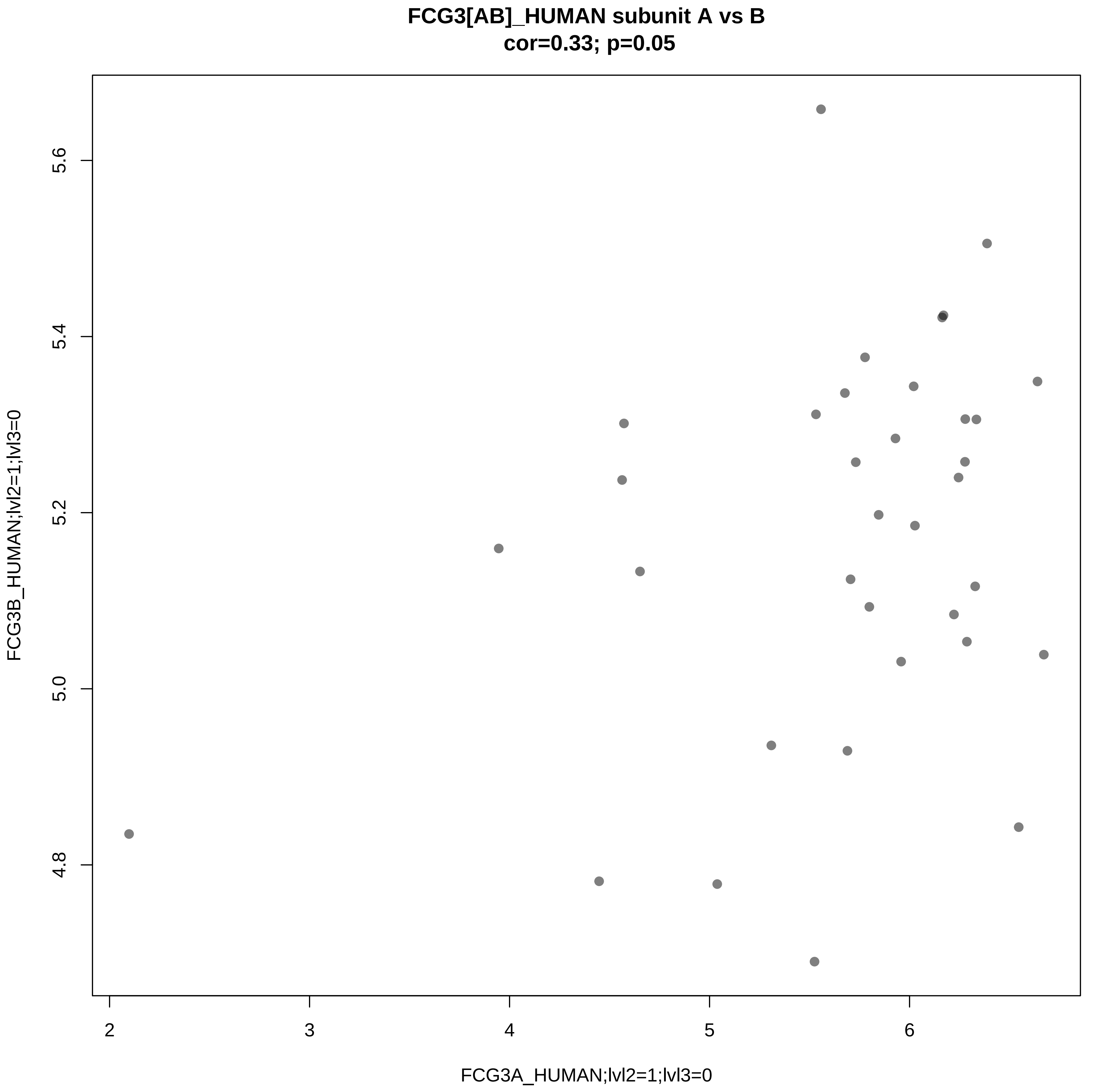
FCG3[AB]_HUMAN subunit A vs B
cor=0.33; p=0.05
FCG3B_HUMAN;lvl2=1;lvl3=0
FCG3A_HUMAN;lvl2=1;lvl3=0
5.6
5.4
5.2
5.0
4.8
2
3
4
5
6

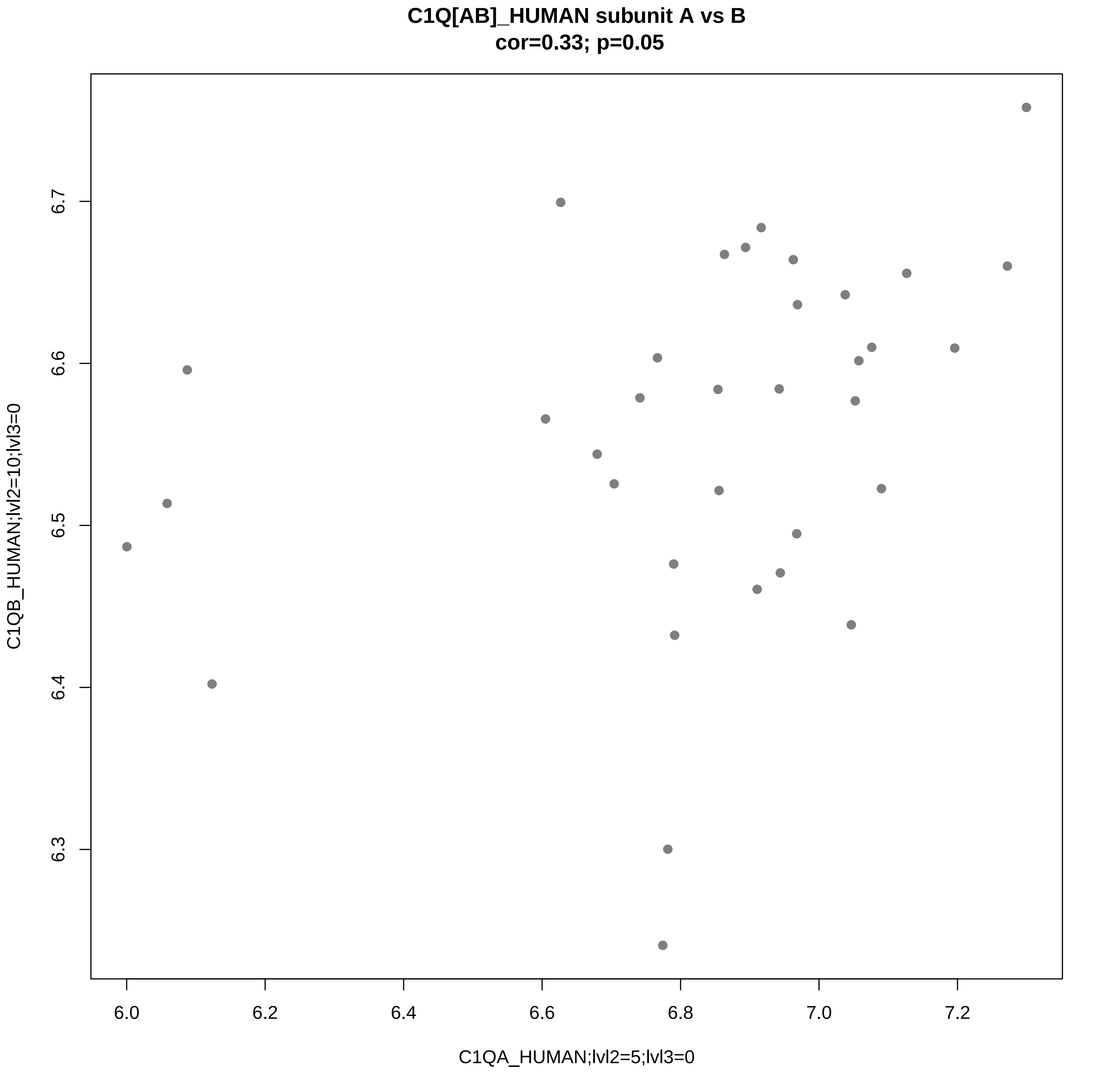
C1Q[AB]_HUMAN subunit A vs B
cor=0.33; p=0.05
6.3
6.4
6.5
6.6
6.7
C1QB_HUMAN;lvl2=10;lvl3=0
6.0
6.2
6.4
6.6
6.8
7.0
7.2
C1QA_HUMAN;lvl2=5;lvl3=0

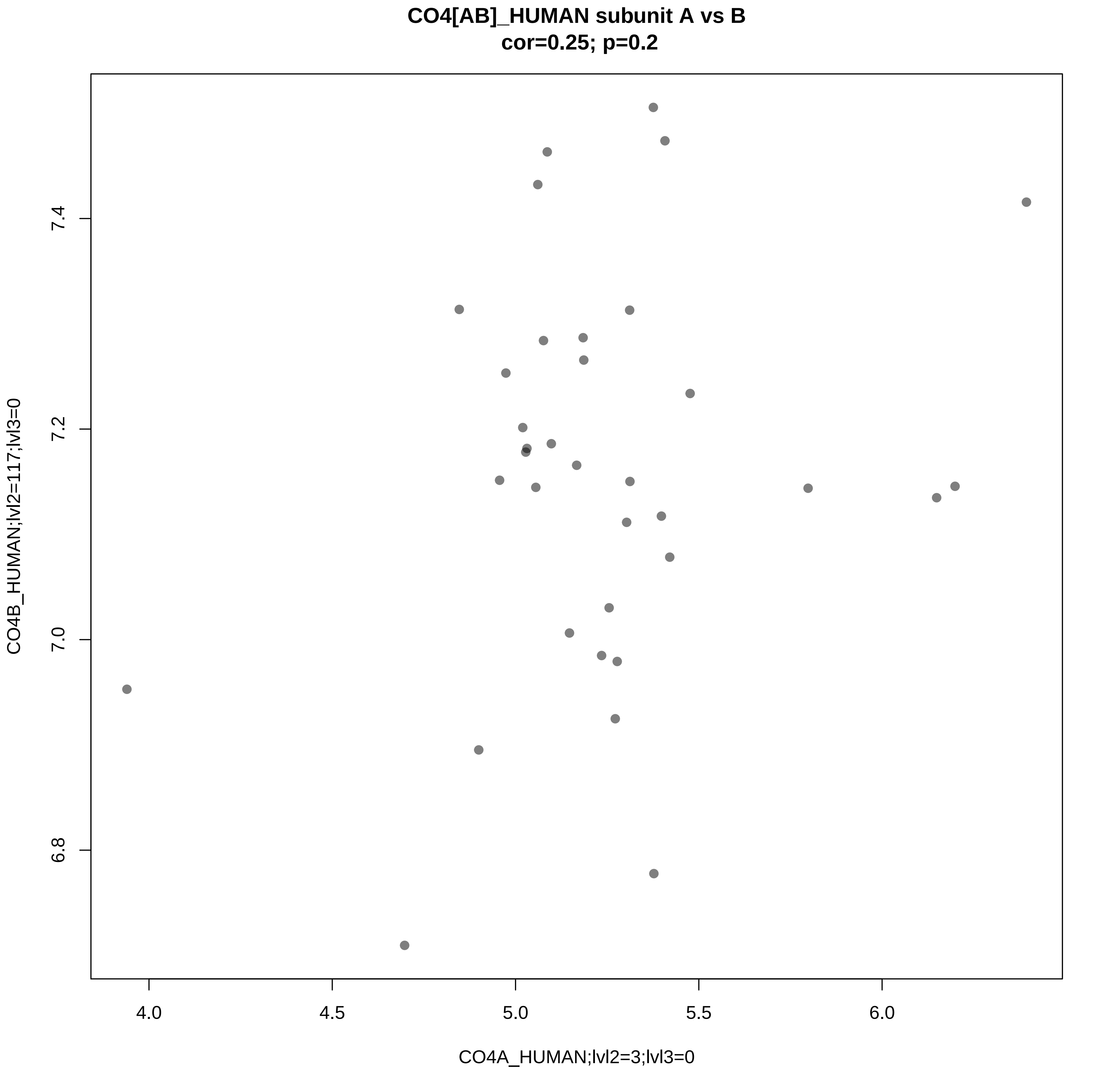

CO4[AB]_HUMAN subunit A vs B
cor=0.25; p=0.2
7.4
7.2
7.0
6.8
CO4B_HUMAN;lvl2=117;lvl3=0
4.0
4.5
5.0
5.5
6.0
CO4A_HUMAN;lvl2=3;lvl3=0

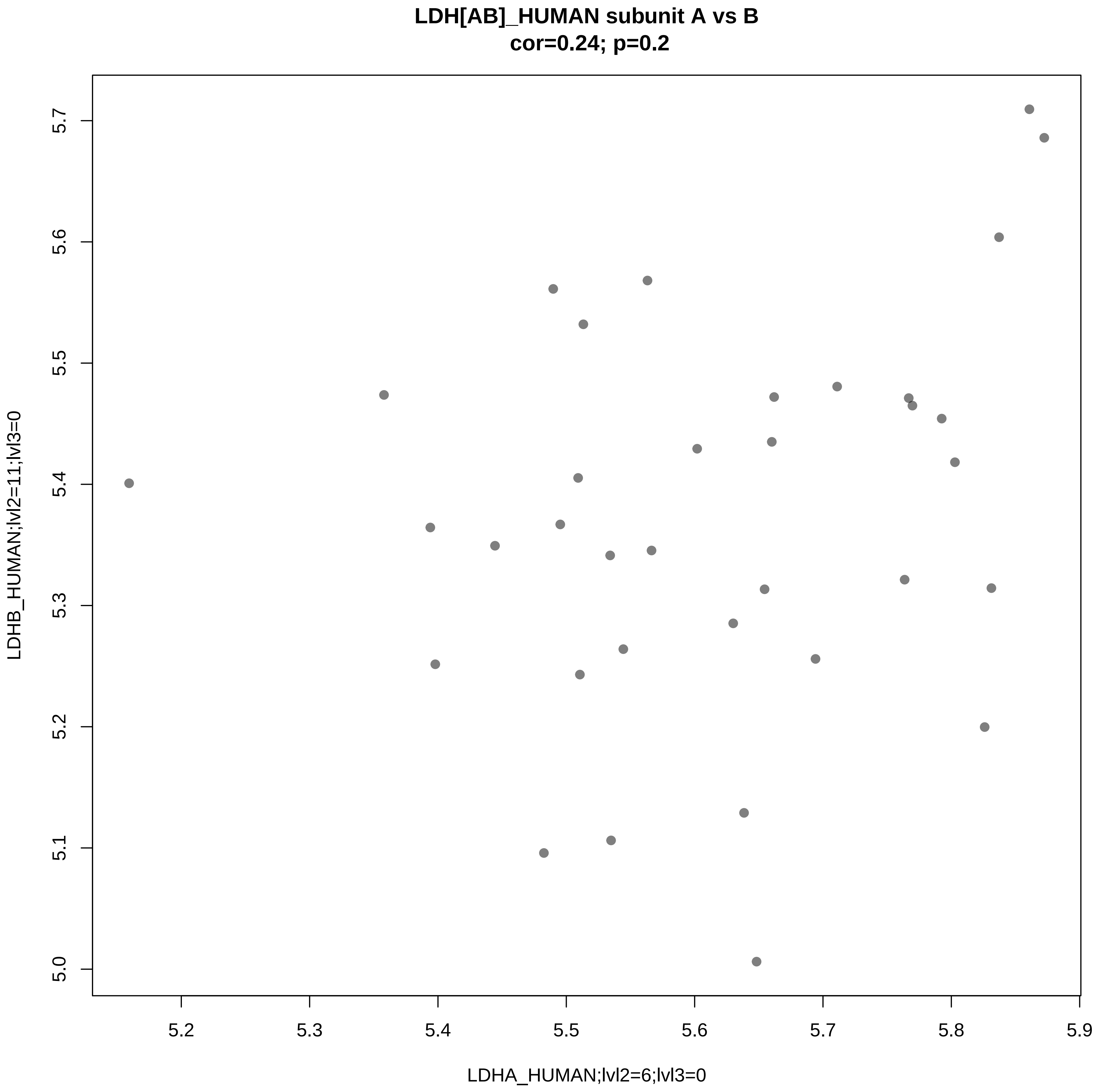

LDH[AB]_HUMAN subunit A vs B
cor=0.24; p=0.2
5.7
5.6
5.5
5.4
5.3
5.2
5.1
5.0
LDHB_HUMAN;lvl2=11;lvl3=0
5.2
5.3
5.4
5.5
5.6
5.7
5.8
5.9
LDHA_HUMAN;lvl2=6;lvl3=0

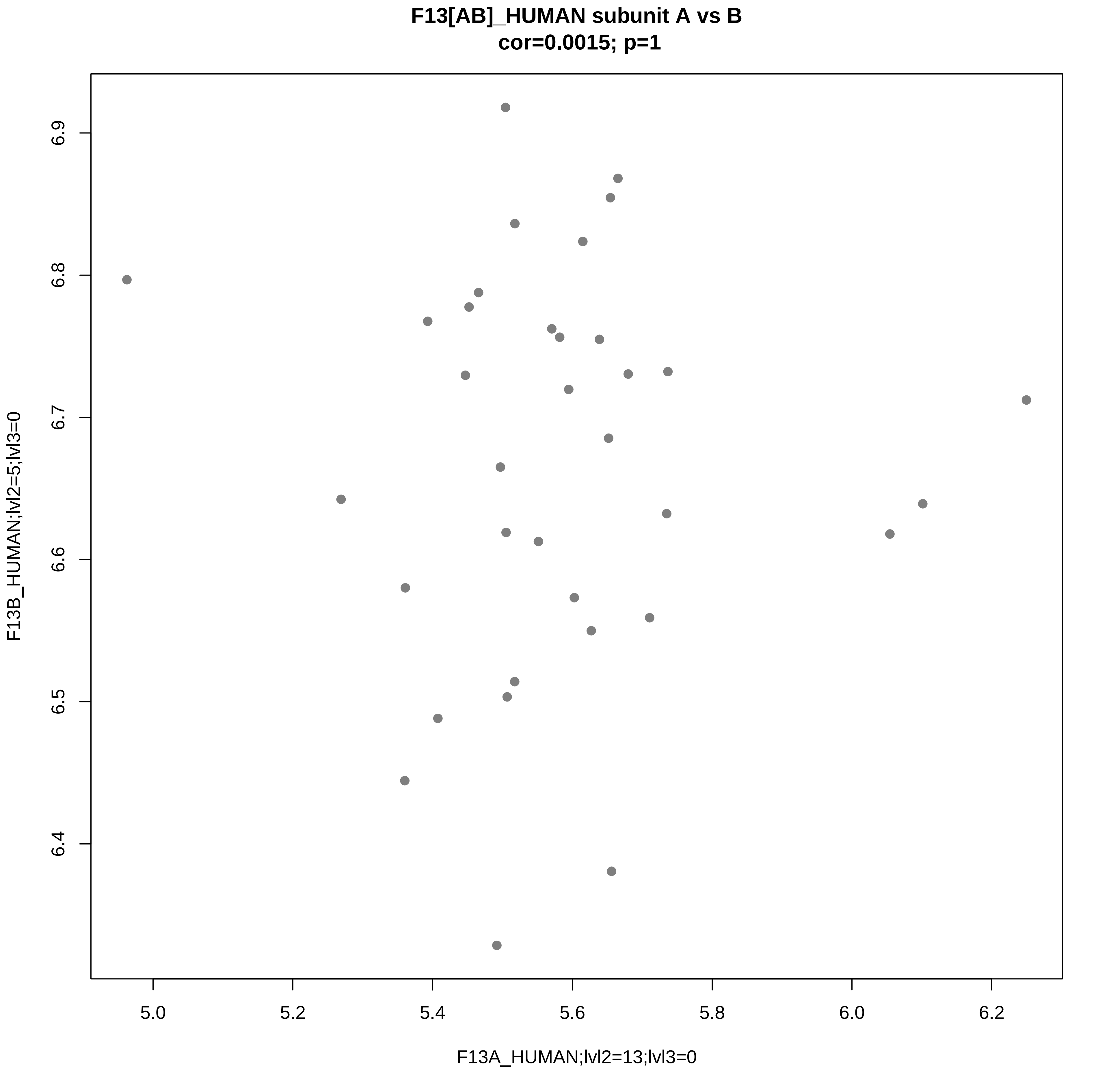
F13[AB]_HUMAN subunit A vs B
cor=0.0015; p=1
6.9
6.8
6.7
6.6
6.5
6.4
F13B_HUMAN;lvl2=5;lvl3=0
5.0
5.2
5.4
5.6
5.8
6.0
6.2
F13A_HUMAN;lvl2=13;lvl3=0

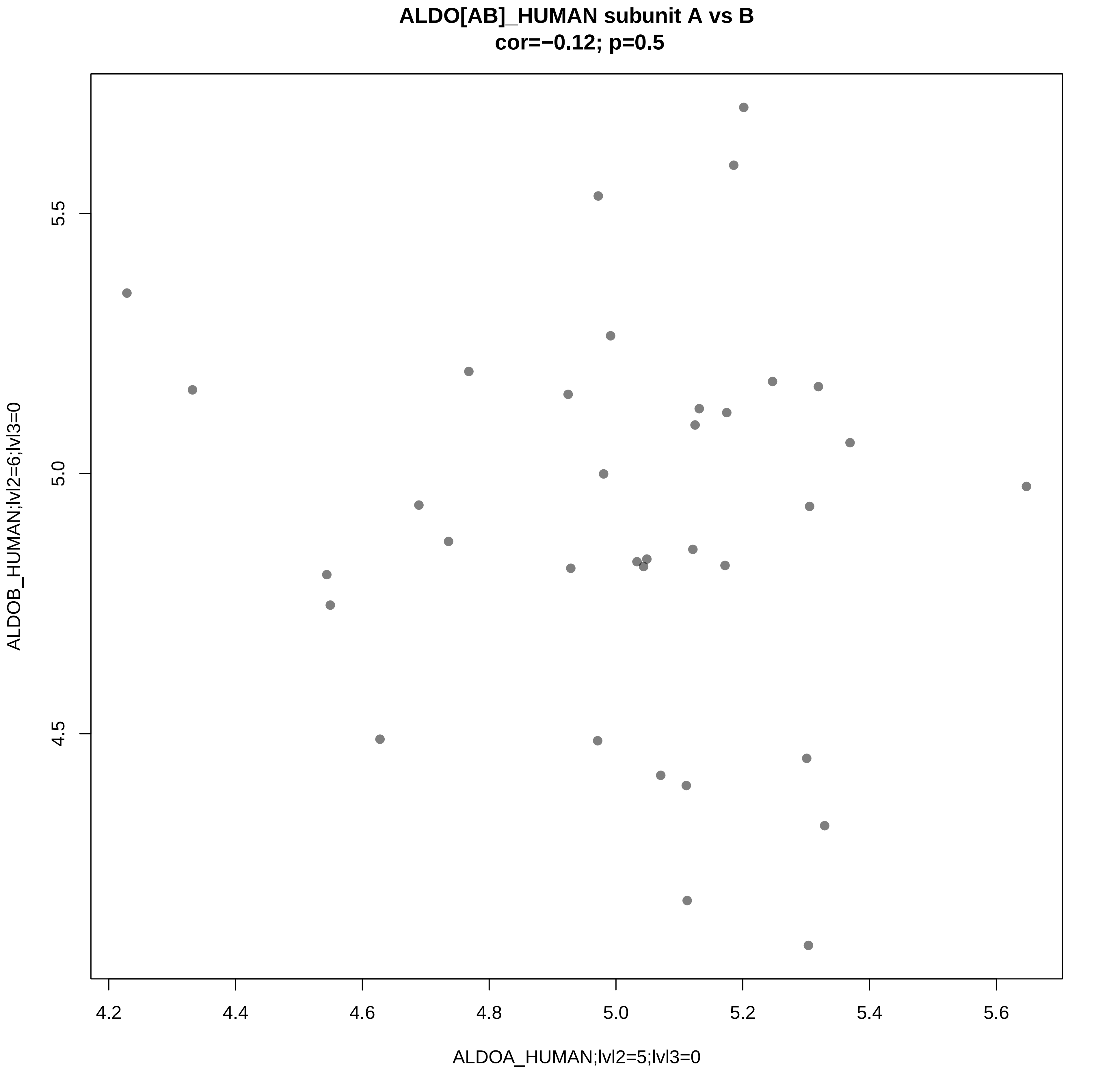
ALDO[AB]_HUMAN subunit A vs B
cor=−0.12; p=0.5
5.5
5.0
4.5
ALDOB_HUMAN;lvl2=6;lvl3=0
4.2
4.4
4.6
4.8
5.0
5.2
5.4
5.6
ALDOA_HUMAN;lvl2=5;lvl3=0
