# Supplementary material for: Severe COVID-19 in pregnancy has a distinct serum profile, including greater complement activation and dysregulation of serum lipids
Source: PLoS One. 2022 Nov 16;17(11):e0276766. doi: 10.1371/journal.pone.0276766 (PMC9668183; doi:10.1371/journal.pone.0276766)
Supplement: Attachment — Submitted filename: response to reviewers.docx [file pone.0276766.s004.docx]

September 11, 2022

Dear Dr. Catherine Mounier and *PLOS One* editorial board,

Thank you so much for the invitation to revise and resubmit our manuscript *Severe COVID-19 in pregnancy has a distinct serum profile, including greater complement activation and dysregulation of serum lipids”*. We believe this has greatly strengthened our manuscript and we are excited about the resubmission as we have done significant changes that have improved the manuscript and created a resource via large supplemental files that we believe will be very useful for future studies.

We have also re-run all analysis with updated software with minimal changes to the results, minor changes in p-values and no changes in pathway enrichment; however, we felt that best to provide the most recent possible data.

You will also notice that we have added many new linear models for interpretation throughout the manuscript and in all supplemental files. Our models now include:

lm1: severeCOVID
lm1b: severeActiveCOVID
lm2a: severeCOVID + age + race
lm2b: severeCOVID + age + race + covid_ga_w + BMI_delivery
lm2c: severeCOVID + age + race + covid_ga_w + med_cond___obesity
lm3: covid_severity_recent
lm4: covid_severity_recent [excluding severe cases] {basically asymptomatic vs non- and post-infection controls}

NEW lm5: covid_status [excluding COVID within 6 weeks of sampling] {basically post-infection mild/moderate vs controls}

The novel piece in the resubmission is that we have a cohort consistent with post-infection controls as we discuss so in essence, we can did an analysis on four groups: control, severe infection, asymptomatic infection, **post-infection** mild/moderate (+one asymptomatic) and this strengthens the statistical power significantly. In our tracked changes version, you will notice that we have updated all the text that was relevant.

We believe the comments from the reviewers were helpful to strengthen our work and happy with the current version. Some of the comments from Reviewer 2 related to antibody, cytokines and immune profiles were not within the scope or objectives of this work especially since we focus on LC-MS and metabolomics, lipidomics and proteomics and cytokines and immune cell profiles are a different technology and isolate. That technology and analysis is part of other work unrelated to serum profiles.

We have gone through journal requirements and addressed each point as well as a point by point reviewer comments below (follows this cover letter).

The study was approved by the Institutional Review Board (UCLA IRB# 20-000579). After patient was identified, participants underwent screening and eligible participants were contacted by a study coordinator by phone or email. Verbal or email consented was obtained. All co-authors have read and approved the manuscript in its current submitted form. Authors AQ and SY are co-founders of, are employed by, and own stock in Dalton Bioanalytics Inc. **This does not alter our adherence to PLOS ONE policies on sharing data and materials**. .All other authors report no conflict of interest.

We appreciate the efforts of the reviewers and editorial staff in evaluating our manuscript revision and look forward to your decision.

Sincerely,

Yalda Afshar, MD, PhD

Division of Maternal-Fetal Medicine

Department of Obstetrics and Gynecology

University of California, Los Angeles

200 Medical Plaza, Suite 430; Los Angeles, CA 90095

(310) 794-8492; YAfshar@mednet.ucla.edu

**Point-by-Point Response:**

Journal Requirements:

When submitting your revision, we need you to address these additional requirements.

**1. Please ensure that your manuscript meets PLOS ONE's style requirements, including those for file naming.** The PLOS ONE style templates can be found a <https://journals.plos.org/plosone/s/file?id=wjVg/PLOSOne_formatting_sample_main_body.pdf>

[https://journals.plos.org/plosone/s/file?id=ba62/PLOSOne_formatting_sample_title_authors_affiliations.pdf](https://urldefense.com/v3/__https:/journals.plos.org/plosone/s/file?id=ba62*PLOSOne_formatting_sample_title_authors_affiliations.pdf__;Lw!!F9wkZZsI-LA!B7Dh0jMBnFkPeXdt9J3tmk2Lr-e3udSxT74-NNz8JQQ67HGy9L4xaoopqR3KCo_hQ7ZapjCRtUciL2qZU6Wlog$)

*Thank you. Confirmed.*

2. Please provide additional details regarding participant consent. In the ethics statement in the Methods and online submission information, please ensure that you have specified what type you obtained (for instance, written or verbal, and if verbal, how it was documented and witnessed). If your study included minors, state whether you obtained consent from parents or guardians. If the need for consent was waived by the ethics committee, please include this information.

*We added more detail in the material and methods. “Pregnant people with COVID-19 infection were actively enrolled at the University of California, Los Angeles between October 1^st^, 2020 and February 28^th^, 2021 through a prospective cohort study evaluating maternal and neonatal outcomes of pregnancies with COVID-19 infection. The study was approved by the Institutional Review Board (UCLA IRB# 20-000579). After patient was identified, participants underwent screening and eligible participants were contacted by a study coordinator by phone or email. Verbal or email consented was obtained. Confirmed COVID-19 infection was defined as being SARS-CoV-2 positive by nasopharyngeal RT-PCR. Eligibility for the study included participants >13 years old, pregnant at the time of enrollment, with necessary clinical data and biospecimen collection during pregnancy. Healthy pregnant controls without COVID-19 infection, defined as a negative SARS-CoV-2 positive nasopharyngeal RT-PCR test, were concurrently recruited.”*

3. Thank you for stating the following in the Competing Interests section:

“AQ and SY are co-founders of, are employed by, and own stock in Dalton Bioanalytics Inc. All other authors report no conflict of interest.” Please confirm that this does not alter your adherence to all PLOS ONE policies on sharing data and materials, by including the following statement: "This does not alter our adherence to  PLOS ONE policies on sharing data and materials.” (as detailed online in our guide for authors [http://journals.plos.org/plosone/s/competing-interests).](https://urldefense.com/v3/__http:/journals.plos.org/plosone/s/competing-interests).__;!!F9wkZZsI-LA!B7Dh0jMBnFkPeXdt9J3tmk2Lr-e3udSxT74-NNz8JQQ67HGy9L4xaoopqR3KCo_hQ7ZapjCRtUciL2rttwLMIQ$)  If there are restrictions on sharing of data and/or materials, please state these. Please note that we cannot proceed with consideration of your article until this information has been declared. Please include your updated Competing Interests statement in your cover letter; we will change the online submission form on your behalf.

*Thank you. We added: “we have no problems with sharing the data for this publication” on behalf of AQ and SY to the cover letter.*

4. Please include captions for your Supporting Information files at the end of your manuscript, and update any in-text citations to match accordingly. Please see our Supporting Information guidelines for more information: [http://journals.plos.org/plosone/s/supporting-information](https://urldefense.com/v3/__http:/journals.plos.org/plosone/s/supporting-information__;!!F9wkZZsI-LA!B7Dh0jMBnFkPeXdt9J3tmk2Lr-e3udSxT74-NNz8JQQ67HGy9L4xaoopqR3KCo_hQ7ZapjCRtUciL2qWbEhcnQ$).

*Done.*

Reviewers' comments:
Review Comments to the Author

Please use the space provided to explain your answers to the questions above. You may also include additional comments for the author, including concerns about dual publication, research ethics, or publication ethics. (Please upload your review as an attachment if it exceeds 20,000 characters)

**Reviewer #1: Severe COVID-19 in pregnancy has a distinct serum profile, including greater complement activation and dysregulation of serum lipids investigated the pathophysiology behind various clinical trajectories in pregnant patients diagnosed with COVID-19 using multi-omics profiling. The information contained in the manuscript is timely and makes a significant contribution to the body of literature related to the COVID-19 pandemic, particularly by presenting data from a less-studies population – pregnant persons. The manuscript is generally well-written; therefore, my comments/suggestions/questions are minimal.**

*Thank you so much for the summary and the thoughtful comments and revisions throughout. We agree that pregnant persons are less studied, and we believe that with incorporation of your edits we have made the manuscript stronger.*

**Line 78: include (ARDS) after syndrome as this acronym is used later in the manuscript**

*Done. Thank you. Updated: “The clinical presentation of Coronavirus Disease 2019 (COVID-19) in pregnancy is variable and can range in severity from asymptomatic to critical illness with acute respiratory distress syndrome (ARDS), multiorgan failure, and in some cases, death”*

**Line 109: Healthy pregnant controls without COVID-19...**

*Corrected. Updated: “Healthy pregnant controls without COVID-19 infection, defined as a negative SARS-CoV-2 positive nasopharyngeal RT-PCR test, were concurrently recruited.”*

**Line 114: moderate, or severe**

*Edited as recommended. Updated: “COVID-19 severity was categorized as asymptomatic, mild, moderate, or severe based on the NIH guidelines”*

**Lines 125-126: This section could be revised for clarity. It’s a bit wordy and repetitious. Sentences should begin with numbers (i.e., 31 different serum samples). Were 2 samples collected? If so, why weren’t comparisons made? Were samples collected from more than 31 COVID-positive participants and the ones used here randomly selected? If so, how many and how was the selection made?**

*Updated: “Peripheral blood specimens were obtained from consented patients at the time of study enrollment, as close to diagnosis of acute COVID-19 infection as possible… Of the 267 available samples, 31 serum samples were chosen based on COVID-19 severity (control, asymptomatic, mild/moderate, and severe) for analyses. Each sample was from a unique participant.”*

**Line 152-158: verb tense changes and paragraph begins with a number.**

*Thank you. We edited to improve the language. Updated: “Serum samples underwent multiomic profiling however a single outlier sample was omitted from further analysis likely due to mis-injection (technical error). Relative intensities were log10 transformed and statistically corrected for the technical effects run order and extraction efficiency (average internal standard intensity).”*

**Line 160: What do you mean by available subject variables? COVID / draw?**

*Updated: “Participant demographic and clinical characteristics were clustered based on pairwise correlation distance to identify potentially important associations.”*

**Line 169: A total of 30 participants… There are also several places where number format is incorrection in this section.**

*We are unsure what exactly this means but made sure on revision that all numbers consistent throughout the paper and we kept formatting consistent. We hope this helps ameliorate this issue.*

**Line 177: COVID-19 severity. There are other areas of inconsistency with this term. Please double check**

*Thank you for catching this consistency. Updated all “COVID” to COVID-19*

**Line 189-190: I am not sure what this sentence is trying to say. It seems something is missing.**

*“Precipitous changes in maternal serum were seen in those with severe COVID-19 infection in pregnancy.”*

**Line 236: I think this sentence is confusing, especially considering whether two samples were collected from participants and whether the samples were compared. If you only looked at one sample, the differences might be a better word than changes. Also, differences between pregnancies with and without COVID-19.**

*Updated: “The purpose of this study was to identify metabolic and proteomic differences seen in pregnancies with and without COVID-19 and evaluate for metabolic and proteomic differences between severity of COVID-19 infection. “*

**Table 1: There are 7 individuals in Race/Ethnicity for the Asymptomatic Group; Why is Gestational Age for 28 participants?**

*Updated table, “2 participants did not deliver within the University of California, Los Angeles Health system and thus were missing birthing outcome variables such as gestational age at delivery.”*

**One last comment: Phosphatidylcholine concentrations change during pregnancy due to increased de novo synthesis in the maternal liver. In general, levels increase, particularly in the 2nd trimester, so trimester of measurement may matter. Additionally, infection, inflammation, etc. lead to sequestration of choline in the liver and alteration of the serum levels. I think acknowledging this in the discussion is important, especially as this study is specific to pregnant women and the Severe group was more likely to be obese.**

*Thank you for this important clarifying point and comment. We totally agree and appreciate the gestational age changes as well as the BMI correlation and as such we control for both of these parameters in the models we present. This is a very important clinically relevant modifier and hence built into our model. You will see that new models that are included consider these changes and are detailed below:*

*lm1: severeCOVID
lm1b: severeActiveCOVID
lm2a: severeCOVID + age + race
lm2b: severeCOVID + age + race + covid_ga_w + BMI_delivery
lm2c: severeCOVID + age + race + covid_ga_w + med_cond___obesity
lm3: covid_severity_recent
lm4: covid_severity_recent [excluding severe cases] {basically asymptomatic vs non- and post-infection controls}*

*lm5: covid_status [excluding COVID within 6 weeks of sampling] {basically post-infection mild/moderate vs controls}*

**Reviewer #2: The impact of COVID-19 on pregnancy is an important topic that needs close attention and thorough study. Thus the selection of the topic is of paramount importance. In the present study, the authors tried to evaluate the changes in the plasma proteins and lipids in those who are pregnant and having COVID-18 infection.**

*Thank you. We agree that pregnant people are understudied and we are thrilled to use this technology, LC-MS, metabolomics, proteomics, and lipidomics, in this population which would supplement the vast work on cytokines and antibody response in pregnant patients.*

**After measuring all the parameters, the authors concluded that Pregnancies with severe COVID-19 demonstrate greater inflammation and complement activation and dysregulation of serum lipids. This altered multiomic expression provides insight into the pathophysiology of severe COVID-19 in pregnancy - but did not provide information about cytokines profile, range of D-dimer and ferritin levels.**

*Our focus is on metabolomics, proteomics, and lipidomics. We discuss below that cytokine measurement is not possible by LC-MS which was the focus of this paper. The measurement of cytokines is generally well below the limits of detection for untargeted, undepleted plasma proteomics experiments (~nanomolar) due to low concentration (~picomolar) and interference of high abundance species (e.g. serum albumin). Even with specialized affinity immuno-capture techniques, limits of detection are in the high picomolar range [Stenken, J. A., & Poschenrieder, A. J. (2015). Bioanalytical chemistry of cytokines–a review. Analytica chimica acta, 853, 95-115.]. Thus, alternative approaches (e.g. immunoassays) are better suited for cytokine measurement. The targets of interest to the reviewer were not detected (ferritin, TNF, IL6, , MIF, IL4, IL10, D-dimer) likely because they are too low in concentration (~picomolar). We did measure fibrinogen chains A, B and G (coagulation/D-dimer related) but these were not associated with COVID-19 status in our study. All this data is available in Supplemental Table 1.*

**Some of the issues are:
1. THe number of patients studied is small for a disease like COVID-19.**

*We agree that having more patients would be great and our cohort has grown significantly. However, to be able to do a single run of LC-MS for each sample costs $2,500 per sample (just for running the sample, no analysis, and no personnel). The cost of the experimental run for this paper was close to $75,000 USD. We believe work like ours with deep phenotyping in Supplemental Tables and clinical correlates (Table 1-2) that we provide are ripe for other researchers to continue to ask more focused questions after we provide an -omics tools here for analysis.*

**2. Definition of severity of COVID-19 need to be provided-such as clinical picture, oxygens aturation levels, cytokines levels, and other**

*COVID-19 severity is standardized by the US FDA and IDSA, among others. We use the standardized definitions throughout. Tables 1 and 2 include details of the clinical characteristics.*

**3. In all the study population, authors could have measured concentrations of antibodies against SARS-CoV-2 -both IgM and IgG levels, how these titers correspond with the clinical picture and other clinical and lab parameters.**

*Briefly here and answered in more detail below. IgG and IgM constant heavy chains are found in the data; however, we realize that the reviewer is likely looking for g/L absolute quant and we only have relative intensity, which is what is done with LC-MS The relative quant for those heavy constant chains are already reported in the results table. The objective here was not related to antibody responses and focus on proteomics, metabolomics, and lipidomics.*

**4. In a study of this type it is mandatory to measure plasma cytokines profile (TNF, IL6, MIF, IL-4 and IL-10) and correlate the same to the clinical picture, antibody titers and outcome of the infection.**

*To answer both comment #3 and #4 which recommend the study of antibodies (#3) and cytokines (#4) these are very interesting molecular mechanistic questions that many groups (including us) are tackling and have published on (see below work that our group has done on antibodies and cytokines):*

*Our publications on this topic*

*Cambou MC, , et al. J Infect Dis. 2022 Sep 9. PMID: 36082433*

*Matsui Y, et al. JCI Insight. 2022 Jun 22;7(12): PMID: 35579965.*

*Foo SS, et al. Cell Rep Med. 2021 Nov 16; PMID: 34723226*

*Collaborators/others publications on this topic*

*Atyeo C, et al Cell. 2021 PMID: 33476549; PMCID: PMC7755577.*

*Flannery DD, et al. JAMA Pediatr. 2021 Jun 1. PMID: 33512440; PMCID: PMC7846944.*

*Edlow AG, et al. JAMA Netw Open. 2020 Dec 1: 33351086; PMCID: PMC7756241.*

*However, the objective in this paper and in our paper was the novelty that it is not related to the SARS-CoV2 antibody response, as we have surrogate of immunoglobulin already measured in our .xls presented but this is NOT specific to SARS CoV2 and we did not feel was relevant*, rather we use a novel technology and methodology capable of profiling proteins, lipids, electrolytes, metabolites, drugs, environmental chemicals, and other molecules in a single assay.

*We agree that plasma cytokines and COVID-19 profiles are interesting as they correlate to clinical picture and outcomes. Our study team has investigated this and some work has been published here (Foo SS, Cambou MC,* ***Mok T****, et al. Cell Rep Med. 2021 Nov 16;2(11):100453. 2021 Oct 27. PMID: 34723226; PMCID: PMC8549189.). However, our objective and methodology are not focused on cytokine evaluation as this requires a very different methodology and here we are interested in something aside from cytokines that have been well studied using proteomic, lipidomics, and metabolomics. The measurement of cytokines is generally well below the limits of detection for untargeted, undepleted plasma proteomics experiments (~nanomolar) due to low concentration (~picomolar) and interference of high abundance species (e.g. serum albumin). Even with specialized affinity immuno-capture techniques, limits of detection are in the high picomolar range [Stenken, J. A., & Poschenrieder, A. J. (2015). Bioanalytical chemistry of cytokines–a review. Analytica chimica acta, 853, 95-115.]. Thus, alternative approaches (e.g. immunoassays) are better suited for cytokine measurement.*

**5. If it is possible measurement of Treg and Teff cells would have been helpful.**

*The study of immune populations of Treg and Teff cells are interesting, but not the focus of this work and actually require a different source material than we used. They require the peripheral mononuclear cells (PBMCs) from heparinized the whole blood isolated, versus the heparinized blood (serum) used in our experimental design to ask what the changes at the level of the proteins, metabolomics, and lipids are The laboratories of Drs. Nardy-Gomez, Franco, Collier, and many others have published on this population in pregnant populations with COVID-1*

*Hsieh LE, et al. J Reprod Immunol. 2022 Feb;149:103464. doi: 10.1016/j.jri.2021.103464. Epub 2021 Dec 11. PMID: 34953325; PMCID: PMC8665650.*

*Garcia-Flores V, et al. Nat Commun. 2022 Jan 18;13(1):320. doi: 10.1038/s41467-021-27745-z. PMID: 35042863; PMCID: PMC8766450.*
